# Post-COVID-19 Multisystem Inflammatory Syndrome in Adults (MIS-A) With Elevated Levels of Soluble Urokinase Plasminogen Activator Receptor (suPAR) Treated With Anakinra: A Case Report

**DOI:** 10.7759/cureus.70848

**Published:** 2024-10-04

**Authors:** Maria Christaki, Valentini Samanidou, Angelos Liontos, Revekka Konstantopoulou, Haralampos Milionis

**Affiliations:** 1 1st Division of Internal Medicine and Infectious Diseases Unit, University Hospital of Ioannina, Faculty of Medicine, University of Ioannina, Ioannina, GRC

**Keywords:** anakinra, interleukin-1 receptor antagonist, mis-a, multi-inflammatory syndrome, supar

## Abstract

The COVID-19 pandemic has brought attention to a newly identified syndrome of multisystem inflammation. This potentially fatal complication of the disease was initially observed in children and later in adults. It affects primarily unvaccinated patients and may manifest within a timeframe of 2-12 weeks following infection. Soluble urokinase plasminogen activator receptor (suPAR), a novel biomarker, highlights the severity of inflammation and the degree of immune system activation. Herein, we report a case of a patient with multisystem inflammatory syndrome in adults (MIS-A) and markedly elevated suPAR levels, successfully treated with interleukin-1 (IL-1) receptor antagonist.

A 59-year-old female was admitted to our hospital due to febrile illness (up to 40°C) with chills, vomiting, non-bloody diarrhea, and abdominal pain for four days prior to her admission. She tested positive for SARS-CoV-2 12 weeks before her presentation. During hospitalization, the patient deteriorated clinically with multiorgan involvement and hemodynamically instability, with concomitant markedly elevated inflammatory markers. Extensive workup with high suPAR levels led to post-COVID-19 MIS-A diagnosis, and treatment with dexamethasone and an interleukin-1 receptor antagonist (IL-1ra), anakinra, was administered.

The subcutaneous injection of anakinra effectively and safely deterred MIS-A. Further research is needed to investigate the role of interleukin-1 inhibitors for the management of this potentially life-threatening condition.

## Introduction

Amidst the COVID-19 pandemic, there have been reports of multisystem inflammatory syndrome in adults (MIS-A). MIS-A is a rare yet potentially life-threatening complication following SARS-CoV-2 infection. High levels of inflammatory cytokines and significant inflammatory activity as shown by elevated values of erythrocyte sedimentation rate (ESR), C-reactive protein (CRP), ferritin, D-dimers, and interleukin-6 (IL-6), together with immunological activation, are characteristics of severe COVID-19 illness [1].

Similar to this, the release of soluble urokinase plasminogen activator receptor (suPAR) from immune cells is increased upon an inflammatory stimulus. This marker, the blood suPAR level, is considered an individual's inflammation and immune activation indicator [2]. Herein, we report a case of a patient with a marked systemic immune activation driven by suPAR levels, who was successfully treated with the interleukin-1 (IL-1) receptor antagonist (IL-1ra) anakinra.

## Case presentation

A 59-year-old female patient was referred to our hospital on March 2022, due to febrile illness up to 40°C with chills, vomiting, non-bloody diarrhea, and abdominal pain for four days prior to her presentation. The patient reported no prior medical history and was otherwise healthy. She tested positive for SARS-CoV-2 on real-time reverse transcriptase polymerase chain reaction (RT-PCR) of nasopharyngeal specimen 12 weeks before. During her illness, COVID-19 was exhibiting only mild symptoms. Of note, the patient was unvaccinated for SARS-CoV-2.

She stated that she did not experience any respiratory symptoms. Upon admission, the patient exhibited vital signs within the following ranges: blood pressure of 95/65 mmHg, body temperature of 400°C, heart rate of 90 beats/minute, respiratory rate of 22 breaths/minute, and oxygen saturation of 97% on room air (fraction of inspired oxygen {FiO_2_}: 21%). The physical examination and the review of other systems yielded normal results. The patient's electrocardiogram revealed sinus tachycardia. The laboratory test results (summarized in Table 1) indicated increased levels of C-reactive protein (CRP), mild thrombocytopenia, and anemia, as well as elevated procalcitonin and ferritin. Arterial blood gas was obtained and indicated respiratory alkalosis with lactate levels within the normal range. Initial chest X-ray results were normal without signs of pleural effusions or consolidations. Abdominal ultrasound revealed ascites and mild hepatosplenomegaly. Blood cultures were obtained and empirical treatment with broad-spectrum antibiotics (ceftriaxone and metronidazole) and fluids intravenously were administered as a potential bacterial gastroenteritis. Despite antibiotics, the patient's clinical status continuously deteriorated.

**Table 1:** Main laboratory data during the days of hospitalization LDH, lactate dehydrogenase; CPK, creatine phosphokinase; CRP, C-reactive protein; suPAR, soluble urokinase plasminogen activator receptor; D1, day 1; D5, day 5; D6, day 6; D11, day 11

	Unit	Normal range	At arrival	D1	D5	D6, after anakinra	D11, discharge
Hemoglobin	g/dL	12-16	10.9	10	10.2	10.8	11.5
White blood cells	10^9^/L	4-9	5.7	7.7	18.0	12.4	11.2
Neutrophils	%	50-78	85	83	89	89	85
Lymphocytes	%	20-45	10	17	5	7	22
Monocytes	%	-	5	4	4	4	6
Platelets	10^9^/L	150-400	150.000	145.000	303.000	411.000	331.000
Erythrocyte sedimentation rate	mm/hour	0-30	44	30	57	33	14
Urea	mg/dL	11-54	38	13	30	39	51
Creatinine	mg/dL	0.6-1.2	0.86	0.79	0.72	0.77	0.75
Alanine transaminase (ALT)	IU/L	10-35	40	39	52	46	79
Aspartate transaminase (AST)	IU/L	10-35	42	27	46	27	31
Amylase	IU/L	0-90	37	37	171	151	137
Phosphatase alkaline	IU/L	30-125	62	62	221	239	105
Troponin T (high sensitivity)	pg/mL	0-11.6	3	3.3	33.1	24.1	<2.3
Interleukin-6	pg/mL	6.4	-	-	6.8	28.3	1.4
Triglyceride	mg/dL	40-175	145	147	204	218	153
Ferritin	ng/mL	11-306.8	-	391	1231	542	494
LDH	U/L	115-230	209	236	319	239	191
Fibrinogen	g/L	200-400	480	648	703	648	429
D-dimers	mg/L	-	2.50	3.68	3.95	2.84	0.49
CPK	IU/L	25-160	43	98	70	38	10
Procalcitonin	ng/mL	-	0.49	0.46	0.47	0.32	0.04
CRP	mg/L	<6	269	382	442	175	13
suPAR	ng/mL	2	-	-	>15	-	10.6

**Figure 1:**
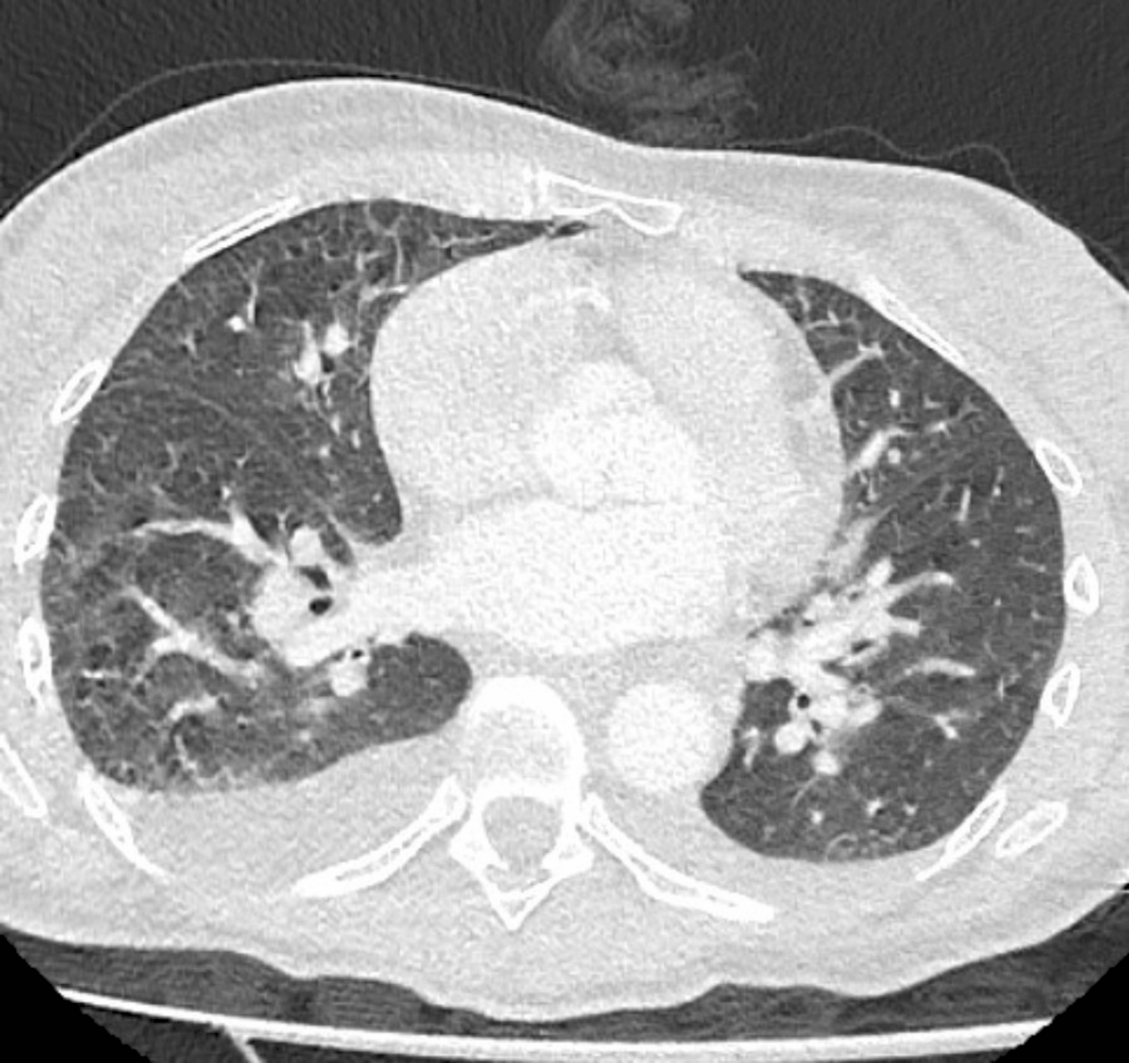
CT of the chest shows bilateral lower lung consolidation with small pleural effusions

The subsequent blood tests revealed elevated liver function tests of aspartate transaminase (AST), alanine transaminase (ALT), alkaline phosphate, and amylase indicating impaired liver function. A CT of the chest, abdomen, and pelvis with intravenous contrast (Figure 1) was performed urgently without any specific findings; a small amount of free intra-abdominal fluid was observed along with reactive lymphadenopathy and bilateral lower lung consolidation with small pleural effusions. However, no identifiable source of sepsis was found.

A comprehensive infectious workup including atypical causes of pneumonia and viral screening including hepatitis B and C panels and HIV resulted in negative. Blood, urine, and stool cultures were also negative. To further investigate the elevated liver function tests, a magnetic resonance cholangiopancreatography was performed. However, no underlying pathology emerged. Based on these findings and due to the lack of response to antibiotics, bacterial sepsis from the respiratory, gastrointestinal, and biliary systems was ruled out. Similarly, screening for autoimmune diseases, including immunoglobulin levels, antinuclear antibodies (ANA), and complement levels, was within normal limits.

**Figure 2:**
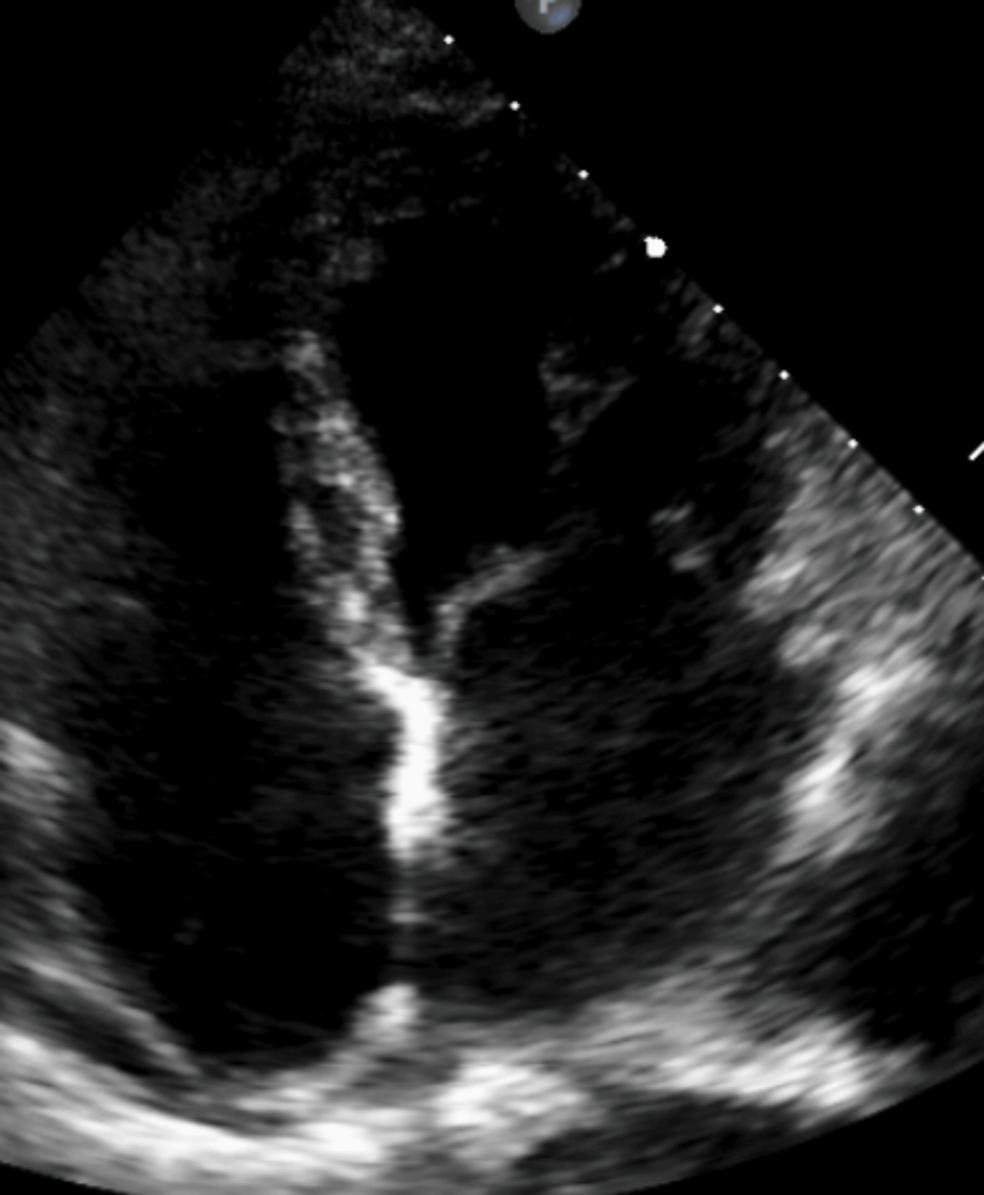
Transthoracic echocardiogram indicated mild pericardial fluid effusion

Repeated blood tests revealed a continued rise in inflammatory markers, with a total white blood cell (WBC) peak of 18 × 109/L, CRP levels of 450 mg/L, and ferritin levels of 1230 ng/mL (Table 1, day 5). By the third day, along with a noticeable increase in inflammatory markers, there was a slight increase in high-sensitivity troponin levels. The onset of acute pulmonary edema followed this rise with symptoms of dyspnea and pleuritic chest pain. A transthoracic echocardiogram was performed, and mild pericardial fluid collection was detected (Figure 2). Consultation with an infectious disease specialist broadened the antibiotic spectrum with meropenem and teicoplanin. Moreover, a colonoscopy was performed, and a colon biopsy showed mild edema and moderate chronic inflammatory lesions without findings indicative of a malignant neoplastic process or autoimmune disease, which ruled out inflammatory bowel disease and ulcerative colitis that were in the differential diagnosis. Hemophagocytic lymphohistiocytosis was excluded due to a lack of diagnostic criteria such as hypofibrinogenemia, cytopenias, and organomegaly, while systemic/allergic drug reactions were also excluded since eosinophils were normal and the patient did not report the initiation of any medication.

Considering the age of >21 years old in a patient with a recent infection from SARS-CoV-2; the constellation of symptoms of continuous fever and hypotension, cardiac involvement, gastrointestinal symptoms, and thrombocytopenia within three days of hospitalization; high inflammatory markers; the deteriorating condition of the patient despite the use of broad-spectrum antibiotics; and no alternative diagnosis more plausible, the possibility of a COVID-19 infection associated with MIS-A was suspected. A third SARS-CoV-2 PCR was negative. However, SARS-CoV-2 serum immunoglobulin G (IgG) antibodies came back as positive. Utilizing RT-PCR and serologic testing for antibodies can assist in diagnosing MIS-A, particularly in those who experienced asymptomatic acute COVID-19. This is because IgG antibodies often become detectable 3-4 weeks following SARS-CoV-2 infection, which aligns with the frequent presentation of MIS-A [3].

Due to the elevated inflammatory markers and the observed plateau despite proper management for infections and her recent SARS-CoV-2 infection, soluble urokinase plasminogen activator receptor (suPAR) was measured and was significantly increased to >15 ng/mL. On day 6, corticosteroid therapy was commenced in the form of intravenous dexamethasone 22 mg daily, an equivalent dose of methylprednisolone 2 mg/kg/day. On day 6, the patient was started on a 10-day course of subcutaneous anakinra 100 mg twice daily, while antibiotics were discontinued on the same day. The patient's vital signs stabilized, and her symptoms improved within 24-48 hours. Concomitantly, laboratory results of the inflammatory markers showed gradual improvement.

**Figure 3:**
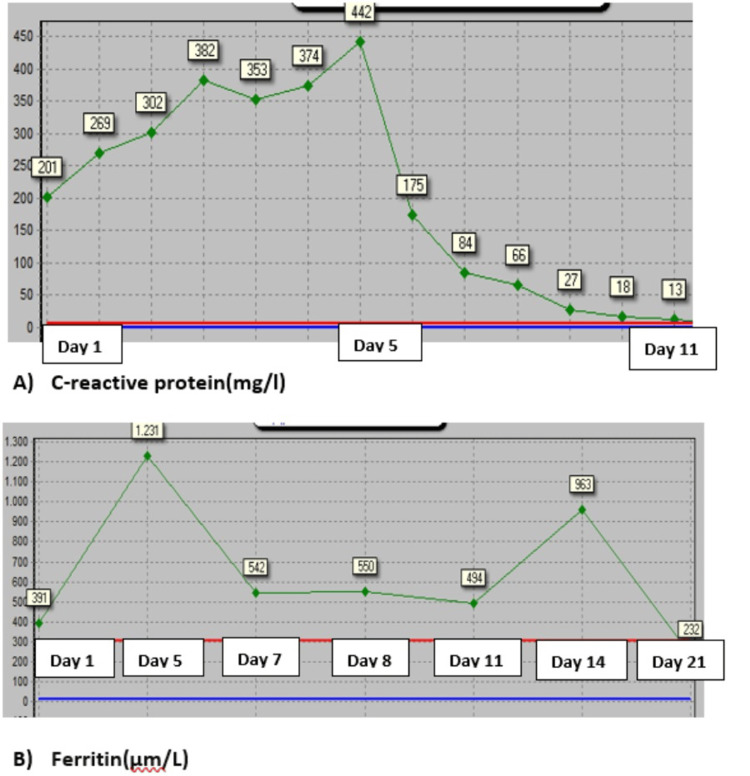
Plot of inflammatory markers The Y-axis represents days of hospitalization, and the X-axis represents laboratory values. (A) C-reactive protein and (B) ferritin

**Figure 4:**
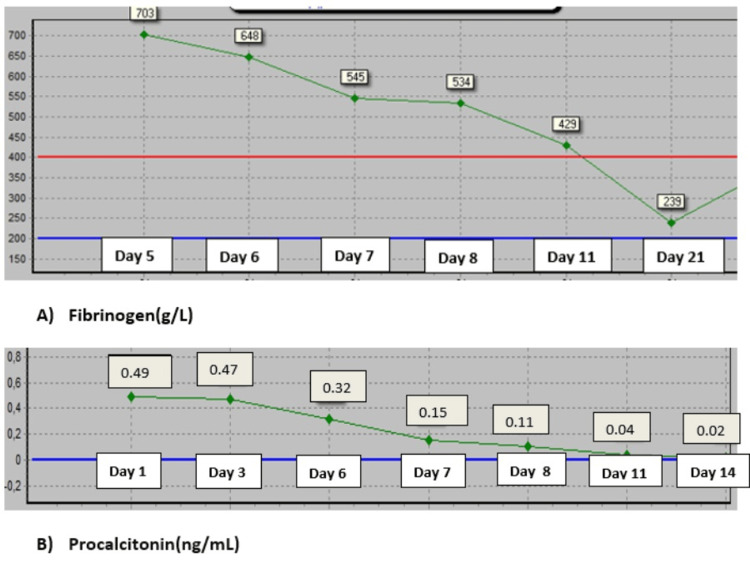
Plot of inflammatory markers The Y-axis represents days of hospitalization, and the X-axis represents laboratory values. (A) Fibrinogen and (B) procalcitonin

Over the following week, all laboratory results normalized. Anakinra was discontinued after 10 days of therapy. She was discharged from the hospital on the 16th day after admission, with a prednisone course starting with 32 mg per day. The dose was tapered by 5 mg every five days. The final diagnosis of the patient was multisystem inflammatory syndrome in adults (MIS-A) according to the CDC 2021 case definition. Subsequent follow-ups were normal (Figures 3, 4).

The administration of the IL-1 receptor antagonist anakinra resulted in a swift enhancement in both the patient's clinical state and metabolic measurements; also, it was found to be safe and tolerable.

## Discussion

MIS-A is a novel inflammatory condition marked by fever and symptoms affecting the cardiovascular, gastrointestinal, mucocutaneous, and neurologic systems. Notably, it is distinct from severe pulmonary illness and typically occurs either concurrently or following a recent positive COVID-19 test. MIS-A is linked to mild respiratory symptoms, hypoxemia, or radiological abnormalities [4].

The etiology of multisystem inflammatory syndrome in adults is hypothesized to be a consequence of a delayed and dysregulated immune response. The exact cause of MIS-A is not completely understood; however, it is believed that the delay in symptoms appearing after a COVID-19 infection is caused by the activation of the adaptive immune response. The possible explanations for malfunction beyond the lungs are endothelial injury and dysregulated innate immune system through the exertion of IL-6, interferon gamma (IFN-γ), IL-10, and tumor necrosis factor (TNF), which consequently leads to T-cell overproduction and B-cell deficiency and lasting cytokine storm [5].

The laboratory findings of MIS-A include pancytopenia, elevated cardiac markers including troponin and brain natriuretic peptide (BNP), increased inflammatory markers, hypoalbuminemia, hypertriglyceridemia, and high liver enzymes [6-8]. MIS-A is also characterized by an overexuberant inflammatory response, as indicated by a substantial increase of inflammatory markers of CRP, ESR, ferritin, and procalcitonin and elevated coagulopathy markers. Other markers of inflammation such as D-dimers, lactate dehydrogenase (LDH), fibrinogen, and IL-6 may also be elevated in MIS-A patients alongside the previously mentioned inflammatory markers but not independently [9]. There are no studies investigating the role of suPAR, a novel biomarker, in patients with MIS-A.

In vitro studies proposed that SARS-CoV-2 could activate NLRP3 inflammasomes, which are potent activators of macrophages, with a marked release of IL-1β, contributing to IL-6 release [10]. Some others have implied that the condition results from a cytokine storm-induced overactive inflammatory response linked to the immunological response to SARS-CoV-2 infection.

The anti-inflammatory effect of corticosteroid therapy is mediated by the release of anti-inflammatory mediators such as IL-10 and by blocking the release of pro-inflammatory/inflammatory mediators including IL-6 and TNF-α, supporting studies in COVID-19 patients that showed decreased values of CRP and IL-6. Several studies on adults with severe COVID-19 pneumonia have shown that blocking IL-1 can effectively reduce the need for oxygen and mechanical ventilation. It also leads to a rapid improvement in the patient's clinical condition and a decrease in inflammatory markers [11]. In our patient, we measured suPAR level before the administration of anakinra, and we found suPAR level extremely elevated at >15 ng/mL. The median suPAR level is around 2 ng/mL and above 6 ng/mL in critically ill patients [12]. A low suPAR level indicates a good prognosis and supports the decision to discharge the patient. suPAR is released during inflammation or immune activation; therefore, its level reflects the extent of immune activation in a patient [13]. Research has shown that suPAR is a biomarker that predicts progression to severe respiratory failure or death in patients with COVID-19. suPAR is increased earlier than other biomarkers including C-reactive protein (CRP), interleukin (IL)-6, ferritin, and D-dimers, and thus, it is important to utilize this biomarker to recognize the patients with MIS-A that would benefit from the early initiation of treatment [14,15].

Measuring suPAR is introducing a personalized treatment approach, because an early increase of suPAR is indicative of the excess release of damage-associated molecular patterns (DAMPs), leading to pro-inflammatory phenomena through the activation of IL-1α/β, which contributes to pathogenic inflammation in COVID-19. Anakinra blocks both IL-1α and IL-1β by blocking their common receptor [16]. Due to the aforementioned elevated levels of suPAR, the already recognized beneficial effect of anakinra in patients with severe COVID-19, and the lack of specific treatment guidelines in patients with MIS-A, we decided to initiate treatment with anakinra in our patient. We believe that suPAR can be utilized to stratify patients with MIS-A who would benefit from the addition of anakinra to corticosteroid therapy.

Anakinra is a recombinant human IL-1 receptor antagonist (IL-1ra) authorized for use in treating rheumatoid arthritis and other illnesses characterized by inflammation and has been found to reduce inflammatory markers such as CRP, ferritin, D-dimers, and IL-6 [17]. Its use showed beneficial effects even in other inflammatory conditions such as secondary hemophagocytic lymphohistiocytosis (sHLH) in pediatric patients [18].

Anakinra has been approved for use in multisystem inflammatory syndrome in children (MIS-C) but has been utilized in limited cases for MIS-A post-COVID-19 infection [19,20]. Furthermore, no recorded case has used suPAR to better select the appropriate candidates for response to treatment with anakinra. Given the improvement in ferritin, CRP, suPAR, and troponin levels with anakinra, we strongly believe that our patient's outcome was influenced by this intervention (Table 1).

When patients show signs of involvement in multiple organs or multiple extrapulmonary symptoms, it is important to consider the possibility of multisystem inflammatory syndrome caused by a secondary SARS-CoV-2 infection. This is particularly relevant if the initial COVID-19 PCR test comes back negative. In such cases, it is crucial to obtain a SARS-CoV-2 IgG test to accurately diagnose the condition.

We aimed to report this case to shed light on the need for early diagnosis and treatment in those patients. The central role of IL-1 in the pathogenesis of the multisystem inflammatory system affirmed the use of anakinra and created a promising perspective. There are no existing guidelines for the management of this condition, but what is clear from our case is that anakinra is an effective treatment that results in the rapid resolution of both clinical and biochemical parameters.

## Conclusions

Increased inflammatory markers and laboratory evidence of a cytokine storm are associated with SARS-CoV-2 infection, which can impact several organs and predispose individuals to life-threatening multisystem inflammation. The early diagnosis and treatment of people with multisystem inflammatory disease are critical. Immunomodulatory therapy should be part of the patient's care. Considering the favorable safety record of anakinra, it is imperative to conduct controlled clinical trials to confirm its efficacy in large groups of patients with MIS-A and define specific guidelines for its utilization.
